# Histopathological findings for prediction of liver cirrhosis and survival in biliary atresia patients after Kasai procedure

**DOI:** 10.1186/s13000-020-00996-y

**Published:** 2020-07-02

**Authors:** Gunadi, Dian Nirmala Sirait, Leila Rakhma Budiarti, Vincentia Meta Widya Paramita, Aditya Rifqi Fauzi, Fiko Ryantono, Dwiki Afandy, Naomi Yoshuantari, Hanggoro Tri Rinonce, Akhmad Makhmudi

**Affiliations:** 1 Pediatric Surgery Division, Department of Surgery, Faculty of Medicine, Public Health and Nursing, Universitas Gadjah Mada/Dr. Sardjito Hospital, Jl. Kesehatan No. 1, Yogyakarta, 55281 Indonesia; 2 Department of Anatomical Pathology, Faculty of Medicine, Public Health and Nursing, Universitas Gadjah Mada/Dr. Sardjito Hospital, Yogyakarta, 55281 Indonesia

**Keywords:** Biliary atresia, Histopathological findings, Liver cirrhosis, Kasai procedure, Prognosis, Patient survival

## Abstract

**Background:**

Without early recognition and Kasai procedure, biliary atresia (BA) results in liver cirrhosis and leads to either transplantation or death at a young age. We aimed to characterize the liver histopathological findings for prediction of cirrhosis and survival in BA patients after Kasai surgery.

**Methods:**

We retrospectively reviewed all histopathological results for BA patients who underwent liver biopsy during Kasai surgery from August 2012 to December 2018 in Dr. Sardjito Hospital, Yogyakarta, Indonesia.

**Results:**

Fifty infants with BA were ascertained in our study, of whom 27 were males and 23 were females. The median age of Kasai procedure was 102.5 days (interquartile range (IQR), 75.75–142.25 days). There were 33 (66%) and 17 (34%) BA patients with and without liver cirrhosis, respectively, while the overall survival was 52%. The patients with a severe bile duct proliferation, severe cholestasis, and severe portal inflammation have a higher risk by 27-, 22-, and 19.3-fold, respectively, to develop liver cirrhosis compared with patients with a moderate/mild bile duct proliferation, moderate/mild/without cholestasis, and moderate/mild portal inflammation, respectively (*p* = 3.6 × 10^− 6^, 5.6 × 10^− 4^, and 1.6 × 10^− 3^, respectively), while the giant cell transformation was not associate with the development of liver cirrhosis (*p* = 0.77). The bile duct proliferation was strongly correlated with cholestasis and portal inflammation (*p* = 7.3 × 10^− 5^ and 2 × 10^− 4^, respectively), and cholestasis was also significantly correlated with portal inflammation (*p* = 0.016). Interestingly, the age at Kasai procedure was strongly associated with the development of liver cirrhosis (*p* = 0.02), but not with the patients’ survival (*p* = 0.33), while the degree of fibrosis and cholestasis were significantly correlated with the patients’ survival, with HR of 3.9 (95% CI = 1.7–9.0; *p* = 0.017) and 3.1 (95% CI = 1.4–7.0; *p* = 0.016), respectively.

**Conclusions:**

Histopathological findings of bile duct proliferation, cholestasis, and portal inflammation can predict the liver cirrhosis development in patients with BA. Furthermore, degree of fibrosis and cholestasis affect the patients’ survival following the Kasai operation.

## Background

Biliary atresia (BA) is characterized by a progressive destructive inflammatory cholangiopathy that affects both the intra- and extrahepatic bile ducts [1, 2]. Its incidence varies among different ethnicities [3–5].

The role of histopathological findings on the prognosis of patients with BA following Kasai operation has been reported with conflicting results [6–8]. Furthermore, BA will lead to liver cirrhosis and results in either transplantation or death at a young age if without early diagnosis and Kasai surgery [1, 2]. Therefore, it is necessary to predict the development of liver cirrhosis and survival in BA patients after Kasai surgery. We aimed to characterize the liver histopathological findings for prediction of cirrhosis and survival in patients with BA following Kasai operation.

## Methods

### Patients

We retrospectively reviewed all histopathological results for patients with BA who underwent liver biopsy during Kasai surgery from August 2012 to December 2018 in our institution, Indonesia.

The study included 55 BA cases, of whom 5 subjects were excluded due to the incomplete data. Fifty patients were included for final analysis (Table 1).

**Table 1:** Baseline characteristics of biliary atresia patients who underwent liver biopsy during Kasai procedure

Characteristics	n (%); median (IQR)
Sex	
▪ Male	27 (54)
▪ Female	23 (46)
Age at Kasai procedure performed (days)	102.5 (75.75–142.25)
Type of biliary atresia (BA)	
▪ 1	2 (4)
▪ 2A	18 (36)
▪ 2B	6 (12)
▪ 3	24 (48)
Pre-operative laboratory findings (normal range)	
▪ Total bilirubin (≤1.0 mg/dL)	10.76 (7.86–13.6)
▪ Direct bilirubin (0–0.2 mg/dL)	8.17 (6.45–11.59)
▪ Aspartate aminotransferase (≤40 U/L)	187 (120.5–242.25)
▪ Alanine aminotransferase (≤41 U/L)	119 (68.75–168.75)
▪ Gamma glutamyl transferase (7–64 U/L)	566 (268.5–946.75)
▪ Alkaline phosphatase (≤462 U/L)	471 (361–648)
▪ Albumin (3.9–4.9 g/dL)	3.55 (3.23–4.14)
▪ International normalized ratio (0.9–1.1)	1.025 (0.94–1.205)
▪ Platelet (150–450 × 10^3^/uL)	270 (218.75–409.25)
Outcomes	
▪ Survived	26 (52)
▪ Died	24 (48)
Causes of death	
▪ Septic shock	14 (56.5)
▪ Hemorrhagic shock	4 (17.4)
▪ Multiple organ dysfunction syndrome	2 (8.7)
▪ Acute respiratory distress syndrome	4 (17.4)

*IQR* interquartile range

The study got an approval by the Ethical Committee of Faculty of Medicine, Public Health and Nursing, Universitas Gadjah Mada/Dr. Sardjito Hospital (KE/FK/528/EC/2015 and KE/FK/0506/EC/2020).

### Histopathological analysis

Histopathological examination was performed by two pathologists at Dr. Sardjito Hospital using hematoxylin and eosin staining. Five histopathological findings were analyzed, including fibrosis, bile duct proliferation, cholestasis, portal inflammation and giant cell transformation, according to previous studies [6, 7, 9, 10].

**Fig. 1:**
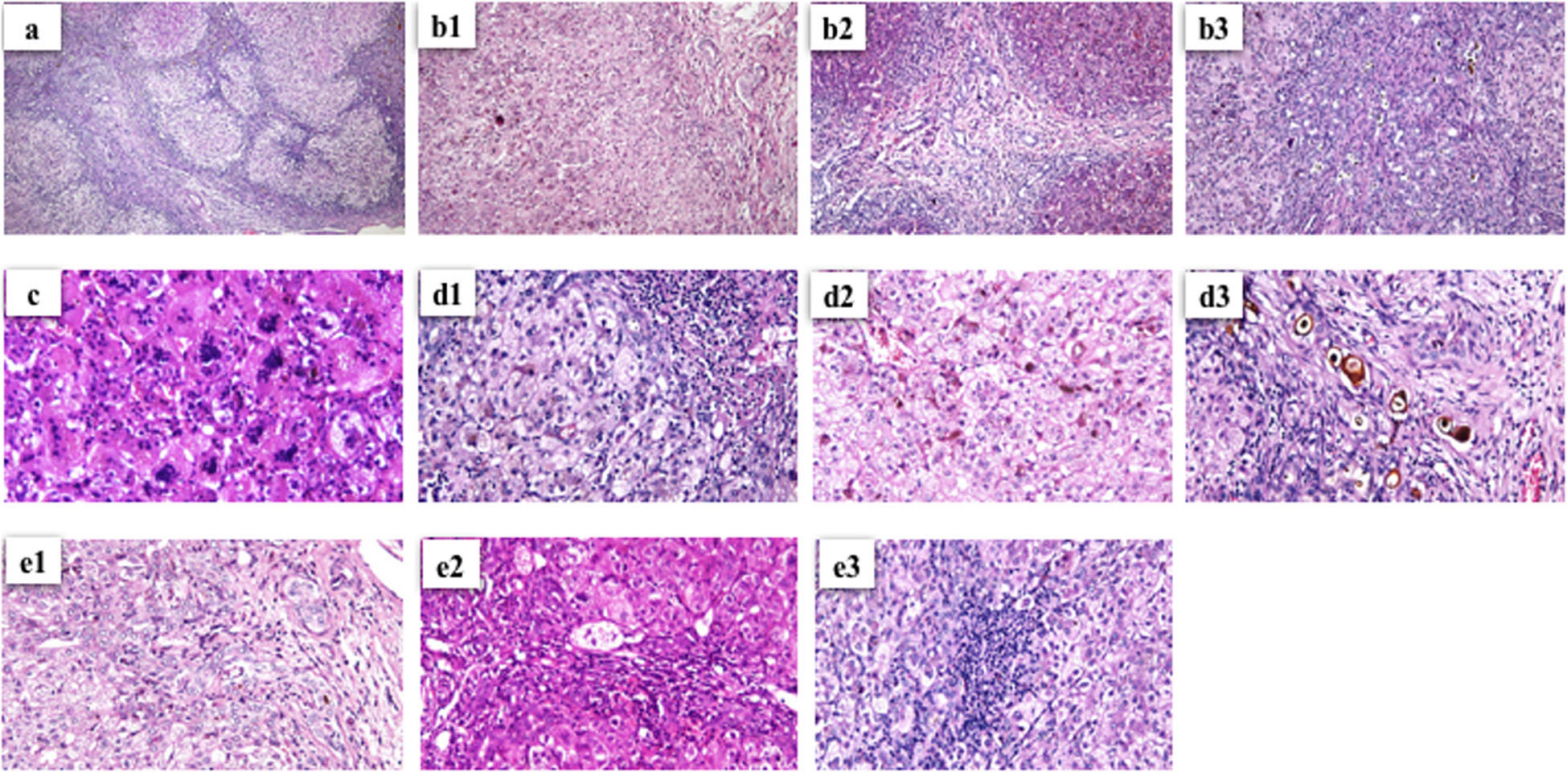
Hematoxylin and eosin staining showed: **a** liver cirrhosis (severe fibrosis) (× 40); **b** bile duct proliferation: 1. mild, 2. moderate, 3. severe (× 40); **c** giant cell transformation (× 400); **d** cholestasis: 1. mild, 2. moderate, 3. severe (× 100); and **e** portal inflammation: 1. mild, 2. moderate, 3. severe (× 100); in BA patients

Fibrosis was classified as follows: 1) mild, fibrosis ranging from portal fibrous expansion to bridging fibrosis encompassing < 50% of portal tracts; 2) moderate, bridging fibrosis with > 50% of portal tracts encompassed without nodular architecture; and 3) severe (cirrhosis), bridging fibrosis with > 50% of portal tracts encompassed and nodular architecture (Fig. 1) [6, 10].

The following grading was used for bile duct proliferation: 1) mild, 5–9 bile ducts per portal tract, 2) moderate, ≥10 bile ducts per portal tract, and 3) severe, ≥10 bile ducts per portal tract and the ducts are elongated attenuated and angulated; whereas cholestasis was classified as: 1) absent, 2) mild, accumulation of bile in centrolobular hepatocytes, 3) moderate, accumulation of bile in centrolobular and periportal hepatocytes or even in portal tracts, and 4) severe, presence of bile infarcts (Fig. 1) [6].

Portal inflammation was categorized as: 1) mild, cells are present in < 1/3 portal tracts, 2) moderate, cells are present in > 1/3–2/3 portal tracts, and 3) severe, dense packing of cells present in > 2/3 portal tracts [6]; while giant cell transformation was assigned as positive and negative (Fig. 1) [7, 9].

### Statistical analysis

We provided the data as number and median (interquartile range, IQR) and utilized the Fischer exact, chi-square, or Mann-Whitney U tests to determine the groups differences. The association between the histopathological findings and the BA patients’ survival was defined by a log-rank test, while the probabilities of patients’ survival following Kasai operation was plotted using Kaplan-Meier curve.

For analysis of association between histopathological findings and liver cirrhosis/survival of BA patients, we combined “mild” and “moderate” categories into one group, i.e. mild/moderate group for fibrosis, bile duct proliferation, and portal inflammation variables, and pooled “absent”, mild”, and “moderate” categories into one group, i.e. absent/mild/moderate group for cholestasis variable.

Kappa values for bile duct proliferation, fibrosis, cholestasis, giant cell, portal inflammation, and cirrhosis were 0.83, 0.97, 0.75, 0.75, 0.82, and 1.0, respectively.

## Results

### Clinical characteristics

The study involved 50 BA patients: 27 males and 23 females. The liver biopsies were conducted at the Kasai procedure, with median age of 102.5 days (interquartile range [IQR], 75.75–142.25) (Table 1). Table 1 provides all pre-operative laboratory findings.

Overall survival of patients with BA was 52%. The causes of the death of BA patients after Kasai are provided in Table 1, while the causes of the death of the non-cirrhotic BA patients (*n* = 7) were multiple organ dysfunction syndrome (*n *= 1), septic shock (*n *= 2), hemorrhagic shock (*n* = 2), and acute respiratory distress syndrome (*n *= 2).

### Association of histopathological findings and liver cirrhosis

First, we analyzed the liver histopathological findings in BA patients and associated them with liver cirrhosis. Among four histopathological findings, bile duct proliferation, cholestasis, and portal inflammation were significantly associated with liver cirrhosis (*p* = 3.6 × 10^− 6^, 5.6 × 10^− 4^, and 1.6 × 10^− 3^, respectively), whereas giant cell transformation was not (*p* = 0.77) (Table 2).

**Table 2:** Association of histopathological findings and liver cirrhosis in BA patients after Kasai procedure

Histopathological findings	Liver cirrhosis		***p***-value	OR (95% CI)
	(+)	(−)		
Bile duct proliferation				
√ Severe	27	3	3.6 × 10^−6^*	27 (5.6–129.1)
√ Moderate/mild	5	15		
Cholestasis				
√ Severe	18	1	5.6 × 10^−4^*	22 (2.6–184.7)
√ Moderate/mild/absent	14	17		
Portal inflammation				
√ Severe	17	1	1.6 × 10^−3^*	19.3 (2.3–162.6)
√ Moderate/mild	15	17		
Giant cell transformation				
√ Positive	17	11	0.77	0.7 (0.2–2.3)
√ Negative	15	7		

*BA* biliary atresia, *OR* odds ratio, *CI* confidence interval

*, *p* < 0.05 is considered statistically significant

Furthermore, the BA patients with severe bile duct proliferation, severe cholestasis, and severe portal inflammation have a higher risk by 27-, 22-, and 19.3-fold, respectively, to develop liver cirrhosis compared with patients with moderate/mild bile duct proliferation, moderate/mild/without cholestasis, and moderate/mild portal inflammation, respectively (Table 2).

### Association among histopathological findings

Next, we determined whether there is an association among histopathological findings. The bile duct proliferation was strongly correlated with cholestasis and portal inflammation (*p* = 7.3 × 10^− 5^ and 2 × 10^− 4^, respectively), and cholestasis was also significantly associated with portal inflammation (*p* = 0.016) (Table 3).

**Table 3:** Association among histopathological findings of liver biopsy in BA patients after Kasai procedure

Histopathological findings	Bile duct proliferation		***p***-value	OR (95% CI)
	Severe	Moderate/mild		
Cholestasis				
√ Severe	18	1	7.3 × 10^−5^*	28.5 (3.4–242.1)
√ Moderate/mild/absent	12	19		
Portal inflammation				
√ Severe	17	1	2 × 10^−4^*	24.8 (2.9–210.5)
√ Moderate/mild	13	19		
Giant cell transformation				
√ Positive	15	13	0.39	0.5 (0.2–1.7)
√ Negative	15	7		
	**Cholestasis**			
	**Severe**	**Moderate/mild/absent**		
Portal inflammation				
√ Severe	11	7	0.016*	4.7 (1.4–16.3)
√ Moderate/mild	8	24		
Giant cell transformation				
√ Positive	8	20	0.15	0.4 (0.1-
√ Negative	11	11		1.3)
	**Portal inflammation**			
	**Severe**	**Moderate/mild**		
Giant cell transformation				
√ Positive	11	17	0.77	1.4 (0.4–4.5)
√ Negative	7	15		

*BA* biliary atresia, *OR* odds ratio, *CI* confidence interval

*, *p* < 0.05 is considered statistically significant

### Association of histopathological findings with age at Kasai procedure and patients’ survival

**Fig. 2:**
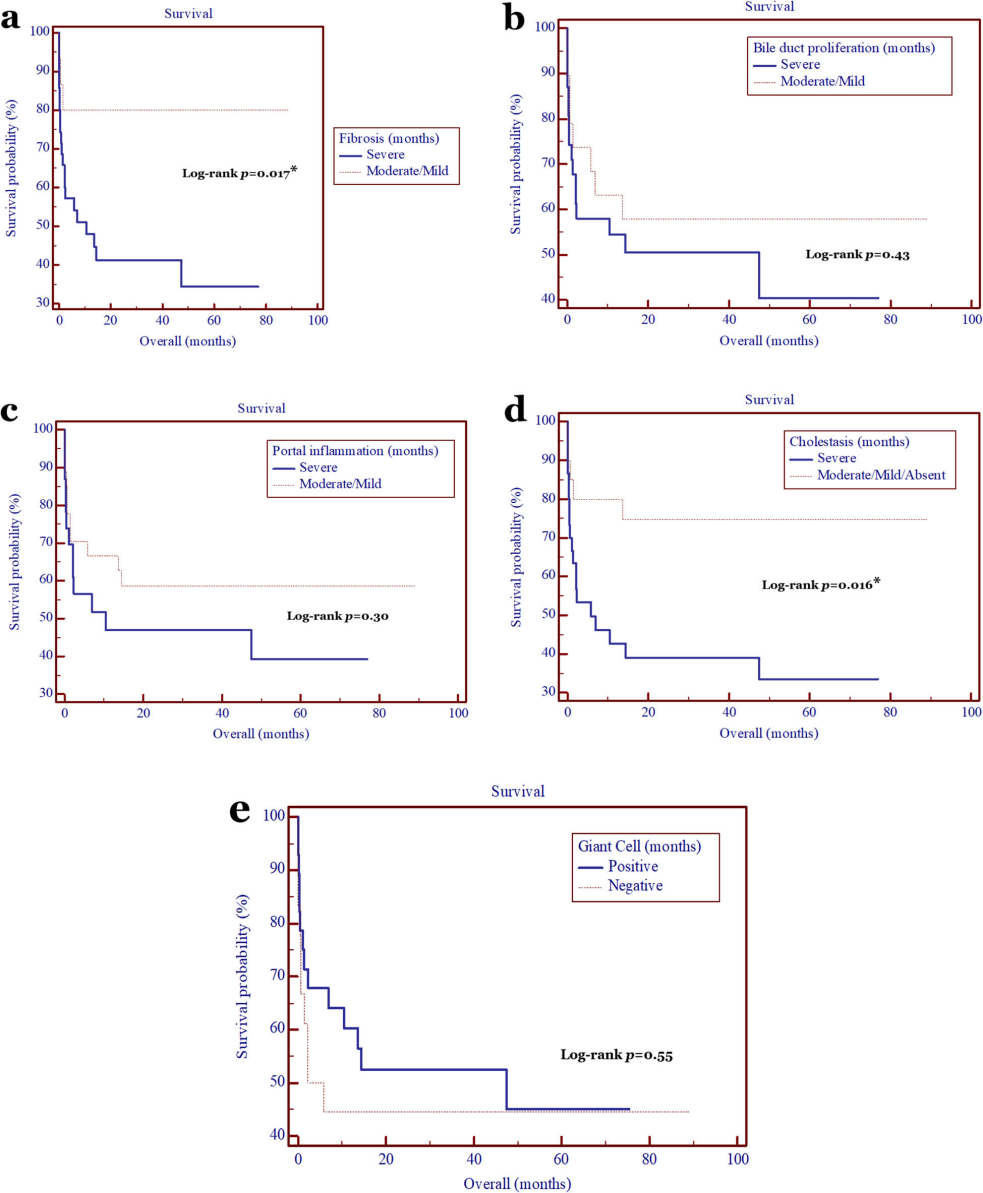
Kaplan-Meier analyses of patients’ survival with BA after Kasai procedure according to the histopathological findings. The degree of fibrosis (**a**) and cholestasis (**d**) were significantly correlated with the patients’ survival, with hazard ratio of 3.9 (95% CI = 1.7–9.0; *p* = 0.017) and 3.1 (95% CI = 1.4–7.0; *p* = 0.016), while bile duct proliferation (**b**), portal inflammation (**c**), and giant cell transformation (**e**) were not (*p* > 0.05)

Subsequently, we correlated the histopathological findings with the age at Kasai procedure and patients’ survival. The age at Kasai procedure was strongly correlated with the liver cirrhosis (*p* = 0.02), bile duct proliferation (*p* = 0.026), and giant cell transformation (*p* = 0.049) (Table 4), while the degree of fibrosis and cholestasis were significantly correlated with the patients’ survival, with HR of 3.9 (95% CI = 1.7–9.0; *p* = 0.017) and 3.1 (95% CI = 1.4–7.0; *p* = 0.016) (Fig. 2).

**Table 4:** Correlation of histopathological findings and age at Kasai procedure performed in BA patients

Histopathological finding	Age at Kasai (days) (median, [IQR])	***p***-value
Bile duct proliferation		0.026*
√ Severe	118 (97–174)	
√ Moderate/mild	86 (72–112)	
Cholestasis		0.2
√ Severe	124 (88.5–184.5)	
√ Moderate/mild/absent	100 (75.5–125.5)	
Portal inflammation		0.21
√ Severe	110 (88.5–176)	
√ Moderate/mild	97 (74–126)	
Giant cell transformation		0.049*
√ Positive	94 (72–113)	
√ Negative	118 (96.5–174)	
Cirrhosis		0.02*
√ Positive	113 (91–171)	
√ Negative	85 (72–97)	

*BA* biliary atresia, *IQR* interquartile range

*, *p* < 0.05 is considered statistically significant

**Fig. 3:**
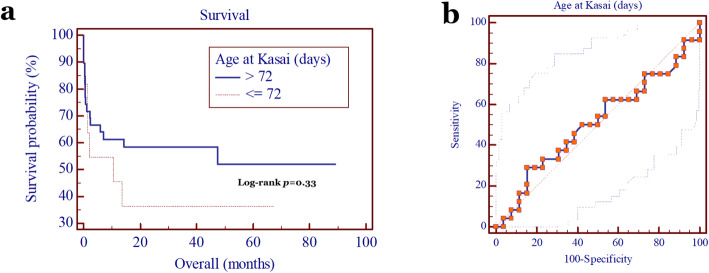
**a** Kaplan-Meier analysis showed that the patients’ survival was not affected by the age when Kasai surgery was performed (*p* = 0.33); **b** ROC curve of age when Kasai surgery was performed with cutoff value of 72 days (sensitivity 29.2%, specificity 84.6%, and area under curve 0.51 [95% CI = 0.36–0.65])

Moreover, there was no significant correlation between age at Kasai surgery and patients’ survival (*p* = 0.33; Fig. 3a), with its cutoff value of 72 days (sensitivity 29.2%, specificity 84.6%, and AUC 0.51 [95% CI = 0.36–0.65] (Fig. 3b).

#### Association of pre-operative laboratory findings and liver cirrhosis

None of pre-operative laboratory findings, including total bilirubin (*p* = 0.79), were associated with the development of liver cirrhosis (Table 5).

**Table 5:** Association of pre-operative laboratory findings and liver cirrhosis in BA patients

Laboratory Findings	Liver Cirrhosis (median, [IQR])		***p***-value
	(+)	(−)	
Total bilirubin (mg/dL)	11.03 (7.72–13.97)	9.88 (8.51–12.55)	0.79
Direct bilirubin (mg/dL)	9.17 (6.85–11.89)	7.79 (7.52–8.85)	0.38
Aspartate aminotransferase (U/L)	212 (157–249)	133 (110–231)	0.07
Alanine aminotransferase (U/L)	117 (72–164)	121 (68–176)	0.94
Gamma glutamyl transferase (U/L)	568 (323–887)	541 (230.5–1002)	0.62
Alkaline phosphatase (U/L)	456 (347.5–681)	526 (425.5–641.5)	0.68
Albumin (g/dL)	3.46 (3.23–3.71)	3.73 (3.36–4.14)	0.33
International normalized ratio	1.01 (0.94–1.32)	1.03 (0.99–1.14)	0.69
Platelet (×10^3^/uL)	254 (220–391)	321 (213–428)	0.73

*BA* biliary atresia, *IQR* interquartile range

## Discussion

Our study is able to find evidence that bile duct proliferation, cholestasis, and portal inflammation are predictor factors for the liver cirrhosis development in patients with BA following Kasai procedure in Indonesia. These findings repeat those reported in previous studies [6, 8, 10], but, there are two novelties in our report: 1) it was performed in Indonesia (versus Indian [6, 8] and Brazilian [10] population); and 2) we associated the histopathological findings with the liver cirrhosis, age at Kasai procedure, and BA patients’ survival (versus the clearance of jaundice [8] and survival [6, 10]). In contrast, Czubkowski et al. [7] concluded that liver histopathological findings have a limited value as prognostic factors for BA patients.

Bile duct proliferation has been shown as a prognostic factor for BA patients. More severe bile duct proliferation has the worst prognosis [6] and increased degree of fibrosis [10]. Our study provides new evidence from a different population that the bile duct proliferation might predict the liver cirrhosis development in patients with BA after Kasai procedure. Furthermore, our findings also support that cholestasis has a prognostic significance for BA patients as reported by Muthukanagarajan et al. [6].

Several studies showed an inconsistent result regarding the role of portal inflammation for prognosis of BA patients [6, 7, 11]. Our result supports the finding that portal inflammation is a prognostic factor for BA patients [11].

Giant cells were seen in 48% of BA patients which is similar with previous studies that ranged from 20 to 50% [12–14]. It has been hypothesized that giant cells are the best indicator for diagnosis of neonatal hepatitis, but not BA [9, 12–14]. Our study also shows that giant cells do not function as a prognostic factor for liver cirrhosis development. These findings are compatible with previous report [7]. However, Azarow et al. [15] revealed that the presence of giant cells is correlated with the failure of the Kasai procedure. These differences might be associated with the subjectivity of histological assessment [7, 16].

Interestingly, the bile duct proliferation revealed a strong association with both cholestasis and portal inflammation and the cholestasis showed a significant association with portal inflammation. Further study is necessary to investigate how they might influence each other and promote the development of liver cirrhosis.

BA will lead to in liver cirrhosis and results in either transplantation or death at a young age if without early diagnosis and Kasai surgery [1, 2]. Thus, the possibility of the liver cirrhosis, especially in BA patients with the severe form of bile duct proliferation, cholestasis, and portal inflammation, even following a Kasai surgery should be clarified during counseling to the families.

It should be noted that we performed the liver biopsy during the Kasai procedure only, therefore, we were unable to find the association between the development of liver cirrhosis and the elapsed time following the surgery, becoming a weakness of our report.

Moreover, the degree of fibrosis and cholestasis were associated with the BA patients’ survival in our cohort after Kasai surgery. Some histopathological features, including fibrosis degree, were strongly associated with the risk of transplantation [17]. Unfortunately, due to the very few cases of pediatric liver transplant in our hospital (*n* = 5), we were unable to analyze the association between histopathological findings and transplantation risk.

The association between age at Kasai procedure and prognosis of BA patients is still controversial. Some studies showed that earlier age of Kasai has a good prognosis for BA patients’ survival [18–20], while other reports did not support this association [21–23]. Our study revealed that age at Kasai procedure had a strong correlation with the development of liver cirrhosis, but not on patients’ survival.

Finally, caution should also be taken when generalizing about these results because this was a mono-institutional study and used a small sample size. These limitations indicate that a multicenter study with a larger sample of cases is further needed to confirm our study.

## Conclusions

Histopathological findings of bile duct proliferation, cholestasis, and portal inflammation can predict the liver cirrhosis development in patients with BA. Furthermore, degree of fibrosis and cholestasis affect the patients’ survival after the Kasai procedure.

## Data Availability

All data generated or analyzed during this study are included in the submission. The raw data are available from the corresponding author on reasonable request.

## Abbreviations

ALT: alanine aminotransferase
ALP: alkaline phosphatase
AST: aspartate aminotransferase
AUC: area under curve
BA: biliary atresia
CI: confidence interval
GGT: gamma glutamyl transferase
HR: hazard ratio
INR: international normalized ratio
IQR: interquartile range
ROC: receiver operating characteristic
